# *Streptomyces lydicamycinicus* sp. nov. and Its Secondary Metabolite Biosynthetic Gene Clusters for Polyketide and Nonribosomal Peptide Compounds

**DOI:** 10.3390/microorganisms8030370

**Published:** 2020-03-06

**Authors:** Hisayuki Komaki, Akira Hosoyama, Yasuhiro Igarashi, Tomohiko Tamura

**Affiliations:** 1 Biological Resource Center, National Institute of Technology and Evaluation (NBRC), 2-5-8 Kazusa-kamatari, Kisarazu, Chiba 292-0818, Japan; hosoyama-akira@nite.go.jp (A.H.); tamura-tomohiko@nite.go.jp (T.T.); 2 Biotechnology Research Center and Department of Biotechnology, Toyama Prefectural University, 5180 Kurokawa, Imizu, Toyama 939-0398, Japan; yas@pu-toyama.ac.jp

**Keywords:** biosynthetic gene cluster, lydicamycin, polyketide, nonribosomal peptide, *Streptomyces*

## Abstract

(1) Background: *Streptomyces* sp. TP-A0598 derived from seawater produces lydicamycin and its congeners. We aimed to investigate its taxonomic status; (2) Methods: A polyphasic approach and whole genome analysis are employed; (3) Results: Strain TP-A0598 contained ll-diaminopimelic acid, glutamic acid, glycine, and alanine in its peptidoglycan. The predominant menaquinones were MK-9(H_6_) and MK-9(H_8_), and the major fatty acids were C_16:0_, *iso*-C_15:0_, *iso*-C_16:0_, and *anteiso*-C_15:0_. *Streptomyces* sp. TP-A0598 showed a 16S rDNA sequence similarity value of 99.93% (1 nucleottide difference) to *Streptomyces angustmyceticus* NRRL B-2347^T^. The digital DNA–DNA hybridisation value between *Streptomyces* sp. TP-A0598 and its closely related type strains was 25%–46%. Differences in phenotypic characteristics between *Streptomyces* sp. TP-A0598 and its phylogenetically closest relative, *S. angustmyceticus* NBRC 3934^T^, suggested strain TP-A0598 to be a novel species. *Streptomyces* sp. TP-A0598 and *S. angustmyceticus* NBRC 3934^T^ harboured nine and 13 biosynthetic gene clusters for polyketides and nonribosomal peptides, respectively, among which only five clusters were shared between them, whereas the others are specific for each strain; and (4) Conclusions: For strain TP-A0598, the name *Streptomyces lydicamycinicus* sp. nov. is proposed; the type strain is TP-A0598^T^ (=NBRC 110027^T^).

## 1. Introduction

The genus *Streptomyces* is the largest taxon within the phylum *Actinobacteria*, and the members are an attractive source of bioactive secondary metabolites. A large number of bioactive compounds have been discovered from them, many of which have been developed into pharmaceuticals and are clinically used [1,2]. Genome analyses of *Streptomyces* strains revealed that each strain has a large and linear chromosome encoding more than 20 secondary metabolite biosynthetic gene clusters (smBGCs), even if it is known to produce only few secondary metabolites. This means that hitherto reported compounds are nothing more than only a part of the secondary metabolites that they can produce. One-half to three-quarters of smBGCs in *Streptomyces* genomes is polyketide synthase (PKS) and nonribosomal peptide synthetase (NRPS) gene clusters [3], suggesting polyketides and nonribosomal peptides are major secondary metabolites in this genus. Type I PKSs and NRPSs are large multifunctional enzymes with various catalytic domains, and the metabolites are synthesized according to the co-linearity rule of assembly lines. Hence, the chemical structures of the polyketide and peptide backbones can be bioinformatically predicted according to domain organizations of the gene clusters [4]. Because polyketides and nonribosomal peptides show various bioactivities, genome mining focused on PKS and NRPS gene clusters often leads to the discovery of new biologically active compounds.

In the search for new anti-methicillin-resistant *Staphylococcus aureus* antibiotics from marine actinomycetes, *Streptomyces* sp. TP-A0598 was isolated from deep sea water and found to produce lydicamycin and its new congeners, TPU-0037-A to TPU-0037-D, of polyketide origin [5]. The biosynthetic gene cluster (BGC) for these compounds was identified through analysis of its genome, and then their biosynthetic pathway was proposed [6]. In this study, we found that *Streptomyces* sp. TP-A0598 is phylogenetically close to *Streptomyces angustmyceticus* with a 16S rDNA sequence similarity of 99.93%. 

Nowadays, 16S rDNA sequence analysis has conventionally been employed to identify each producer at genus-level but the producers are rarely identified at species-level in natural product studies. However, it is important to classify antibiotic producers at the species level because relationships between taxonomic species and their secondary metabolites are useful information to prioritize strains as a screening source for bioactive compounds. Thus, we investigated the taxonomic status of strain TP-A0598 using a polyphasic approach and then surveyed PKS and NRPS gene clusters in the genome. We also discuss the similarity and difference of these smBGCs among taxonomically close strains.

## 2. Materials and Methods

### 2.1. Strains

*Streptomyces* sp. TP-A0598 was isolated as previously described [5] and is available from the NBRC Culture Collection (NBRC-CC) as NBRC 110027 [6]. *S. angustmyceticus* NBRC 3934^T^ was obtained from the NBRC-CC.

### 2.2. Phenotypic and Chemotaxonomic Characterization

To determine the optimal temperature and pH for growth, the strain was incubated for 7 days in Bacto^TM^ Tryptic Soy Broth (TSB; Becton, Dickinson and Company, Sparks, MD, USA) at 5 °C, 10 °C, 20 °C, 25 °C, 28 °C, 37 °C, and 45 °C and at pH 3 to pH 13, respectively. Growth in various concentrations of NaCl was also examined after 7 days of incubation in TSB. Chemotaxonomic experiments were conducted on the basis of a previous report [7]. Physiological and biochemical characteristics were evaluated using API ZYM, API Coryne, and API 50CH Biochemical Test Kits (bioMérieux, Marcy I’Etoile, France) according to the manufacturer’s instructions. Assimilation of carbon sources at a final concentration of 1% (w/v) was tested using ISP 9 agar as the basal medium according to Pridham and Gottlieb [8].

### 2.3. Phylogenetic Analysis Based on 16S rDNA Sequences

The 16S rDNA sequence was determined as previously described [6], and EzBioCloud was used in the similarity analysis to type strains of valid species [9]. Phylogenetic trees were constructed using the neighbour-joining [10] and the maximum likelihood methods with Kimura 2-parameter model [11] via ClustalX, MEGA X [12], and NJplot.

### 2.4. Genome Analysis

Genomic DNA preparation and the whole genome shotgun sequencing of *S. angustmyceticus* NBRC 3934^T^ were performed as described in a previous report [6]. The sequence redundancy for the draft genome was 103-fold. The draft genome sequence was composed of 43 scaffolds with a total size of 8.09 Mb. Draft genome sequence of *S. angustmyceticus* NBRC 3934^T^ has been available in GenBank/ENA/DDBJ under accession numbers BLAG01000001-BLAG01000046. Digital DNA–DNA hybridization (DDH) between the draft genome sequences of *S. angustmyceticus* NBRC 3934^T^ and *Streptomyces* sp. TP-A0598 (BBNO01000001-BBNO01000020) was conducted using Formula 2 of Genome-to-Genome Distance Calculator [13]. Average nucleotide identity (ANI) values were calculated using the ANI Calculator (www.ezbiocloud.net/tools/ani) [14]. PKS and NRPS gene clusters were analyzed as described in a previous report [15]. Rates of PKS and NRPS gene clusters conserved between two strains (RC) were calculated as follows: RC (%) = *C*_ab_ × 2 × (A + B)^−1^ × 100, where *A*, *B*, and *C*_ab_ are the numbers of PKS and NRPS gene clusters in strain A, in strain B, and conserved between strains A and B, respectively.

## 3. Results

### 3.1. Classification of Streptomyces sp. TP-A0598

The whole-cell hydrolysate of *Streptomyces* sp. TP-A0598 contained ll-diaminopimelic acid, glutamic acid, glycine and alanine. The major menaquinone was MK-9(H_6_) and MK-9(H_8_), whose contents were 53% and 36%, respectively. MK-9(H_2_) and MK-9(H_4_) were also observed as minor components (each at <10%). The major cellular fatty acids (>10% of the total) were C_16:0_, *iso*-C_15:0_, *iso*-C_16:0_, and *anteiso*-C_15:0_. These chemotaxonomic data corresponded to the feature of the genus *Streptomyces*.

**Figure 1:**
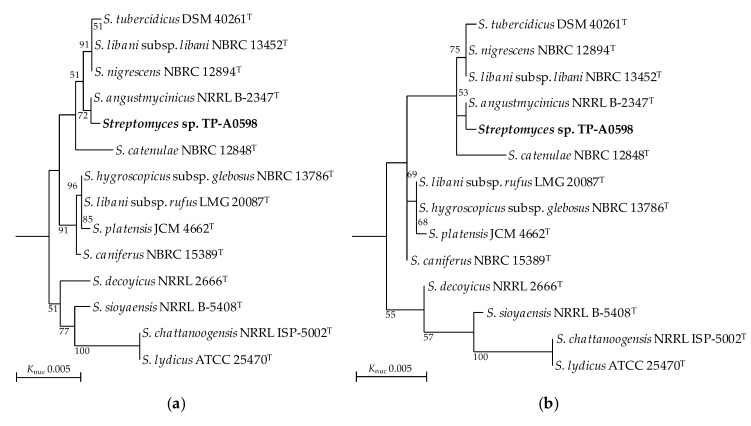
Phylogenetic trees of *Streptomyces* sp. TP-A0598 and type strains of the related species based on 16S rDNA sequences. (**a**) The neighbour-joining method; (**b**) the maximum likelihood method. Numbers on branches are the confidence limits estimated using bootstrap analysis with 1000 replicates; only values higher than 50% are at branching points. Strains with >99.0% similarities to *Streptomyces* sp. TP-A0598 are shown. The sequence of *Embleya scabrispora* NBRC 100760^T^ (AB249946) was used as an outgroup. The accession numbers of *Streptomyces* strains are as follows: *S. angustmycinicus* NRRL B-2347^T^, MUAY01000275; *S. tubercidicus* DSM 40261^T^, AJ621612; *S. libani* subsp. *libani* NBRC 13452^T^, AB184414; *S. libani* subsp. *libani* NBRC 13452^T^, AB184414; *S. catenulae* NBRC 12848^T^, AB184191; *S. catenulae* NBRC 12848^T^, AB184191; *S. hygroscopicus* subsp. *glebosus* NBRC 13786^T^, AB184479; *S. libani* subsp. *rufus* LMG 20087^T^, AJ781351; *S. platensis* JCM 4662^T^, AB045882; *S. decoyicus* NRRL 2666^T^, LGUU01000106; *S. sioyaensis* NRRL B-5408^T^, DQ026654; *S. chattanoogensis* NRRL ISP-5002^T^, LGKG01000206; *S. lydicus* ATCC 25470^T^, RDTD01000009.

*Streptomyces* sp. TP-A0598 showed a 16S rDNA sequence similarity of 99.93% to *S. angustmyceticus* NRRL B-2347^T^ (1450/1451, 1 nucleotide [nt] difference) as the closest species and formed a monophyletic clade with *S. angustmyceticus* in the phylogenetic trees based on 16S rDNA sequences (Figure 1). Phylogenetically close species were *Streptomyces nigrescens*, *Streptomyces libani* subsp. *libani*, and *Streptomyces tubercidicus*, which showed similarities of 99.79%, 99.79% (3 nt difference), and 99.72% (4 nt diffrenece) to strain TP-A0598, respectively. *S. nigrescens* and *S. libani* subsp. *libani* have been reported to be the same species [16]. These three strains of two species formed an independent clade, of which the position is close to that of TP-A0598. *Streptomyces catenulae* NBRC 12848^T^ was also close to the two clades, although the bootstrap values are not high. Digital DDH indicated that DNA–DNA relatedness between *Streptomyces* sp. TP-A0598 and the four type strains are 25%–46%, which is much lower than the cut-off point of 70% recommended for the assignment of bacteria strains to the same genomic species [17]. ANI values between *Streptomyces* sp. TP-A0598 and the four type strains were 82.1–92.3%, whose values are also below the recommended threshold for species delineation (95%–96%) [18]. These results suggest *Streptomyces* sp. TP-A0598 to be a novel genomospecies.

Next, we characterized the strain by comparing it with its phylogenetically closest relative, *S. angustmyceticus* NBRC 3934^T^. Strain TP-A0598 formed light yellow or vivid yellow substrate mycelia and white to grey aerial mycelia and produced a vivid yellow soluble pigment when cultured on trypticase soy agar. The morphological features cultured on the other agar medium are summarized in Table 1. The pH and temperature ranges for growth were pH 5–11 and 15–37 °C, respectively. The optimum temperature was 25–28 °C. At 37 °C, abundant sporulation was observed. The strain grew in the presence of 0%–5% NaCl (w/v). Strain TP-A0598 showed morphological, chemotaxonomic, physiological, and biochemical features different from those of *S. angustmyceticus* NBRC 3934^T^ (Table 2).

### 3.2. PKS and NRPS Gene Clusters of Streptomyces sp. TP-A0598 and S. angustmyceticus NBRC 3934^T^

*Streptomyces* sp. TP-A0598 harbours two type I PKS (*t1pks-1*, *t1pks-2*), two type II PKS (*t2pks-1*, *t2pks-2*), one type III PKS (*t3pks-1*), two NRPS (*nrps-1*, *nrps-2*), and two hybrid PKS/NRPS (*pks/nrps-1*, *pks/nrps-2*) gene clusters in the genome as listed in Table 3. *Pks/nrps-1* is already reported as the BGC for lydicamycins [6]. *T2pks-1*, *t2pks-2* and *t3pks-1* were identified as BGCs for spore pigment, oxytetracycline (*oxy*) and tetrahydroxynaphthalene (THN) (*rpp*), respectively. The other clusters are orphan, whose products have not been specified by previous studies. We predict the putative backbone for metabolites produced by *nrps-1*, *nrps-2,* and *pks/nrps-2* as shown in Table 3 based on their module organisations and substrates of adenylation domains [4,15].

The genome of *S. angustmyceticus* NBRC 3934^T^ encoded four type I PKS (*t1pks-1, t1pks-2, t1pks-3, t1pks-4*), two type II PKS (*t2pks-1, t2pks-3*), one type III PKS (*t3pks-1*), four NRPS (*nrps-1, nrps-3, nrps-4, nrps-5*) and two hybrid PKS/NRPS (*nrps-3, nrps-4*), as listed in Table 4. The sequences of *t1pks-3* and *t1pks-4* gene clusters could not be completely determined because their sequences were divided into three and seven scaffolds, respectively. However, all the open reading frames (ORFs) of *t1pks-3* and *t1pks-4* showed high amino acid sequence similarities to TsnB [19] (96%–98%) and ScaP [20] (89%–97%), respectively. Therefore, these gene clusters were considered as BGCs for trichostatin A and caniferolides, respectively. *T2pks-1*, *t2pks-3*, and *t3pks-1* were identified as BGCs for spore pigment, trioxacarcin, and THN (*rpp*), respectively. *Pks/nrps-3* showed similar gene organisation to that of BGC for guadinomine (*gdn*) [21] but did not encode all *gdn* genes. Hence, we predicted the product to be partial. As the other gene clusters were orphan, we predicted the backbone as shown in Table 4. *Nrps-4* and *nrps-5* were not be able to be completely sequenced.

**Figure 2:**
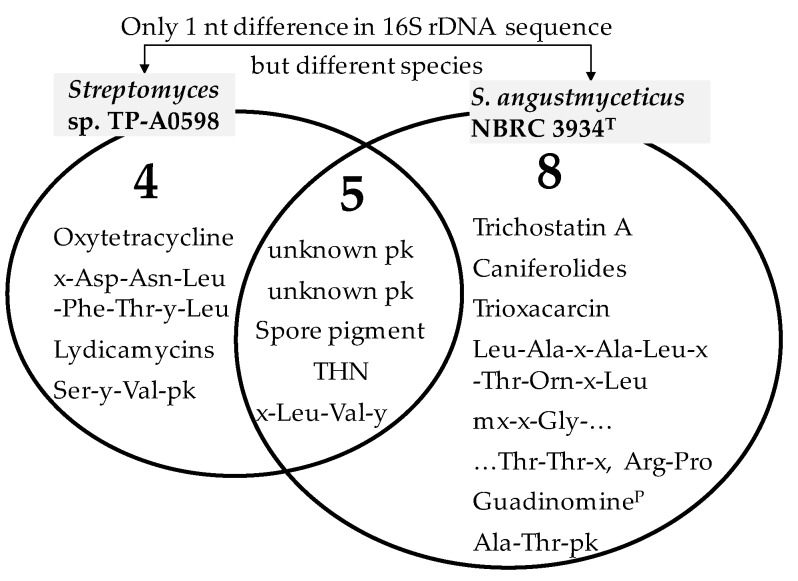
Venn diagram showing the putative compounds derived from PKS and NRPS pathways of phylogenetically close *Streptomyces* sp. TP-A0598 and *Streptomyces angustmyceticus* NBRC 3934^T^. Five are conserved between the two strains, whereas four and eight are specific to *Streptomyces* sp. TP-A0598 and *S. angustmyceticus* NBRC 3934^T^, respectively. pk, moiety derived from PKS pathway; THN, tetrahydroxynaphthalene; x, unidentified amino acid; y, unknown building block; mx, methyl-amino acid; ^P^, partial.

Among the PKS and NRPS gene clusters found in their genomes, five (*t1pks-1*, *t1pks-2*, *t2pks-1*, *t3pks-1*, and *nrps-1*, highlighted in boldface in Table 3 and Table 4) were conserved between the two strains, whereas four (*t2pks-2*, *nrps-2*, *pks/nrps-1*, *pks/nrps-2*) and eight (*t1pks-3*, *t1pks-4*, *t2pks-3*, *nrps-3*, *nrps-4, nrps-5*, *pks/nrps-3*, *pks/nrps-4*) were specific in *Streptomyces* sp. TP-A0598 and *S. angustmyceticus* NBRC 3934^T^, respectively. These numbers and the putative products are summarized in Figure 2. As *Streptomyces* sp. TP-A0598 harbours nine gene clusters, five of nine (56%) were the same as those of *S. angustmyceticus* NBRC 3934^T^ whereas four of nine (44%) were specific to *Streptomyces* sp. TP-A0598. Similarly, because *S. angustmyceticus* NBRC 3934^T^ possesses 13 gene clusters, five of 13 (38%) were the same as those of *Streptomyces* sp. TP-A0598, whereas eight of 13 (62%) are specific to *S. angustmyceticus* NBRC 3934^T^. On average, PKS and NRPS gene clusters conserved between these two strains occupy 45%. We putatively define the rate as RC (rate of PKS and NRPS gene clusters conserved between two strains) for the discussion below.

## 4. Discussion

The difference in 16S rDNA sequences between *Streptomyces* sp. TP-A0598 and *S. angustmyceticus* NBRC 3934^T^ was only 1 bp. Unexpectedly, however, the two strains were classified into different species, and *Streptomyces* sp. TP-A0598 was revealed to be a novel species in this study. Therefore, here, we propose the name *Streptomyces lydicamycinicus* sp. nov. for strain TP-A0598. The description is given below this section.

**Figure 3:**
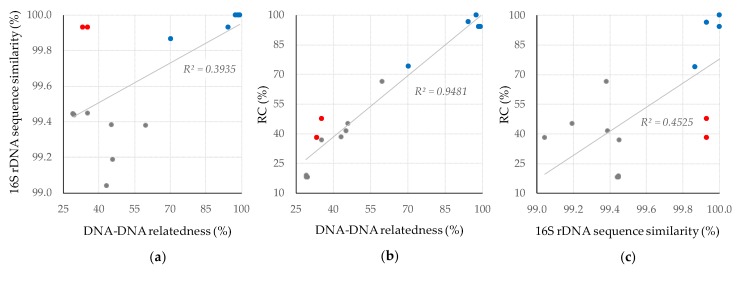
Relationship among 16S rDNA sequence similarity, DNA–DNA relatedness, and rate of PKS and NRPS gene clusters conserved (RC) between *Streptomyces* strains. (**a**) Relationship between 16S rDNA sequence similarity and DNA–DNA relatedness; (**b**) Relationship between RC and DNA–DNA relatedness; (**c**) Relationship between RC and 16S rDNA sequence similarity. RC was calculated as stated in the Materials and Methods section. Blue, the same species (or the same genomospecies); red, different species with 16S rDNA sequence similarity of >99.9%; grey, different species with 16S rDNA sequence similarity of <99.5%. Data were taken from the following strain pairs: blue from *Streptomyces* sp. TP-A0882/*Streptomyces diastaticus* subsp. *ardesiacus* NBRC 15402^T^, *Streptomyces rubrogriseus* NBRC 15455^T^/*Streptomyces violaceoruber* A3(2) [15], *Streptomyces* sp. NBRC 14016/*Streptomyces* sp. NBRC 13827, *Streptomyces* sp. NBRC 14016/*Streptomyces* sp. NBRC 13840 and *Streptomyces* sp. NBRC 13827/*Streptomyces* sp. NBRC 13840 [24]; red from *Streptomyces* sp. TP-A0598/*S. angustmyceticus* NBRC 3934^T^ [this study] and *Streptomyces* sp. CHI39/*S. fragilis* NBRC 12862^T^ [23]; grey from *Streptomyces* sp. TP-A0598/*Streptomyces platensis* DSM 40041^T^, *S. angustmyceticus* NBRC 3934^T^/*S. platensis* DSM 40041^T^ [unpublished], *Streptomyces* sp. TP-A0882/*Streptomyces coelicoflavus* NBRC 15399^T^, *Streptomyces* sp. TP-A0882/*S. rubrogriseus* NBRC 15455^T^, *S. rubrogriseus* NBRC 15455^T^/*S. coelicoflavus* NBRC 15399^T^ [15], *Streptomyces* sp. NBRC 14016/*Streptomyces albospinus* NBRC 13846^T^, *Streptomyces* sp. NBRC 13827/*S. albospinus* NBRC 13846^T^, and *Streptomyces* sp. NBRC 13840/*S. albospinus* NBRC 13846^T^ [24].

Analysis of 16S rDNA sequences is conventionally used as a primary method to identify strains at the genus level. Recently, the similarity of 99.0% was proposed as a boundary to discriminate species in actinobacteria classification [22]. However, a 16S rDNA sequence similarity of >99.0% does not necessarily guarantee that both strains belong to the same species. For example, we have recently classified two strains with completely identical 16S rDNA sequences into two different species [7]. In contrast, there are also cases in which strains showing a difference of 1 nt in their 16S rDNA sequence are classified into the same species [15]. These examples support the notion that 16S rDNA sequences do not have enough resolution to provide definitive identification [22]. For species-level identification, DNA–DNA relatedness provides a clear-cut criterion for bacterial classification [13,16]. We have investigated DNA–DNA relatedness based on whole genome sequences between *Streptomyces* strains with >99% similarities in 16S rDNA sequences [7,15,23,24]. The relationship between 16S rDNA sequence similarity and DNA–DNA relatedness is shown in Figure 3a. Proportional relation may be observed but the correlation coefficient is not high (*R*^2^ = 0.3935), and many strains (grey and red dots) are identified as different species even though their 16S rDNA sequence similarities are >99.0% [7,15,22].

In our previous reports [15,23,24,25], we introduced two hypotheses: (i) Strains belonging to the same species harbour very similar sets of smBGCs; (ii) Strains belonging to different species share only a limited number of smBGCs and each the strain has many species-specific ones. Hypothesis (ii) was supported by the present study even though the 16S rDNA sequence similarity between the two strains is 99.93%. We showed the relationship between DNA–DNA relatedness and the rate of PKS and NRPS gene clusters conserved between *Streptomyces* strains (RC) with >99.0% similarities in 16S rDNA sequences. The relationship is clearly proportional, with a correlation coefficient (*R*^2^) of 0.9481 (Figure 3b). A DNA–DNA relatedness value of 70% is the borderline level at which one can discriminate the same or different species in bacteria [17]. Strains within the same species (blue dot) do not show low RC values, whereas strains classified to different species do not show high RC values. These facts strongly support hypotheses (i) and (ii), described above, respectively. The idea that there is no relation between species classification and secondary metabolites is still widespread. There are two possible reasons why this idea has taken root and continues to persist: (a) only an (or a very limited number of) actually produced secondary metabolite has been investigated and genome information-based comprehensive analyses have not been conducted; (b) the classification of used strains is inaccurate and/or based on only 16S rDNA sequences [26,27,28,29,30,31,32]. If we had focused only on 16S rDNA sequence similarities, we would never have found out about the clear proportionality between phylogenetic distance and RC (Figure 3c). To obtain enough proof of concepts to verify our two hypotheses, we are continuing similar analyses on accurate classification and smBGCs for other phylogenetically close strains in the genus *Streptomyces*.

## 5. Description of *S. lydicamycinicus* sp. nov.

*Streptomyces lydicamycinicus* (lydi’ca.mi.ci’ni.cus. N. L. n. lydicamycin, an antibiotic; L. masc. suffix *-icus* adjectival suffix used with various meanings; N.L. masc. adj. *lydicamycinicus* related to lydicamycin, referring to the ability of the organism to produce lydicamycin and its congeners).

The data were taken from previous reports [5,6] and from this study. Aerobic and Gram stain-positive. Forms light yellow or vivid yellow substrate mycelia and white to grey aerial mycelia. Soluble pigment is yellow to reddish brown. Forms spiral spore chains and the spores were cylindrical, 0.5 × 0.9 μm in size, having a warty surface. Phenotypic characteristics when grown on agar media are shown in Table 1. Grows at 15–37 °C (optimal 25–28 °C), at pH 5–11 (optimal 6–9), and up to 5% NaCl (optimal 0%), but growths at 10°C and pH 11 are considered weak. d-Fructose, d-glucose, d-mannitol, d-raffinose, d-sucrose, and inositol are utilized as a sole carbon source. Assimilation of d-xylose and l-arabinose are weak. Produces acid from *N*-acethylglucosamine, d-adonitol, d-arabitol, d-fructose, d-galactose, gentiobiose, glycerol, glycogen, d-glucose, inositol, d-mannitol, d-mannose, d-melibiose, methyl-α-d-glucopyranoside, methyl-α-d-mannopyranoside, d-raffinose, d-ribose, d-sorbitol, starch, d-sucrose, and d-trehalose. Acid productions from amygdalin, arbutin, esculin ferric citrate, salicin, and xylitol are weak. Does not produce acid from the other sugars in API 50CH. In the API ZYM and API Coryne assays, *N*-acetylglucosaminidase, acid phosphatase, alkaline phosphatase, β-galactosidase, α-glucosidase, leucine aminopeptidase and phosphohydrolase are positive; *N*-acetyl-β-glucosaminidase, cystine aminopeptidase, esterase (C-4), esterase lipase (C-8), pyrrolidonyl arylamidase, and valine aminopeptidase are weakly positive; the other 11 enzymes are negative. Weakly ferments glucose, mannitol and ribose, but not glycogen, lactose, maltose, sucrose, and d-xylose. Negative for nitrate reduction. Hydrolyses gelatin but not esculin and urea. Catalase activity is positive. Oxidase activity is negative. Produces lydicamycin and TPU-0037-A to TPU-0037-D. The predominant menaquinones are MK-9(H_6_) and MK-9(H_8_); MK-9(H_2_) and MK-9(H_4_) are minor components. The major cellular fatty acids are C_16:0_, *iso*-C_15:0_, *iso*-C_16:0_, and *anteiso*-C_15:0_. The DNA G+C content of the type strain is 71.0 mol%.

The type strain is TP-A0598^T^ (=NBRC 110027^T^), isolated from seawater collected in Namerikawa, Toyama, Japan.

## Figures and Tables

**Table 1:** Cultural characteristics of *Streptomyces* sp. TP-0598 and the type strain of its closest phylogenetic neighbour.

Med.		*Streptomyces* sp. TP-A0598	*Streptomyces angustmyceticus* NBRC 3934^T^
ISP 2	SM	++, Vivid yellow	++, Moderate yellow to moderate yellowish brown
	AM	+, Light bluish grey	+, Yellowish grey to dark reddish brown
	SP	Moderate reddish brown	Moderate reddish brown
ISP 3	SM	+, Moderate yellowish brown to moderate reddish brown	±, Moderate yellowish brown
	AM	+, Light yellowish brown to dark greyish red	+, Light olive grey to olive grey
	SP	Light to moderate yellowish brown	Moderate reddish brown
ISP 4	SM	±, Vivid yellow	++, Pale yellow or moderate yellow
	AM	+, Light bluish grey	+, Light olive grey to dark reddish brown
	SP	–	Moderate yellowish brown
ISP 5	SM	+, Light yellow	++, Moderate yellow
	AM	+, White	+, Light bluish grey
	SP	–	Moderate yellowish brown
ISP 6	SM	+, Pale yellow	+, Pale yellow
	AM	–	+, White
	SP	–	–
ISP 7	SM	+, Pale yellow to moderate yellow	++, Moderate yellow
	AM	+, White to light bluish grey	+, White to light bluish grey
	SP	Moderate yellow	Moderate brown
266	SM	+, Moderate yellow to strong brown	++, Moderate yellowish brown to moderate reddish brown
	AM	+, Light bluish grey	+, Light bluish grey
	SP	Deep orange	Moderate brown
228	SM	+, Strong brown	++, Moderate yellowish brown to moderate reddish brown
	AM	+, Light bluish grey to light brownish grey	+, White to greyish blue
	SP	Deep orange	Moderate brown

Med., agar medium; SM, substrate mycelium; AM, aerial mycelium; SP, soluble pigment; 266, Yeast extract-starch; 228, Bennett’s; –, not observed. Growth of mycelium was scored using ++ (good), + (normal) or ± (slight).

**Table 2:** Characteristics different between *Streptomyces* sp. TP-A0598 and the type strain of its closest phylogenetic neighbour.

Characteristic	*Streptomyces* sp. TP-A0598	*S. angustmyceticus* NBRC 3934^T^
Morphological		
Aerial mycelium *	White to grey	White to black
Substrate mycelium *	Light yellow or vivid yellow	Moderate yellow
Soluble pigment	Yellow to Reddish brown	–
Chemotaxonomic		
Major fatty acid (%)	C_16:0_ (23), *iso*-C_15:0_ (12), *iso*-C_16:0_ (12), *anteiso*-C_15:0_ (11)	*iso*-C_16:0_ (31), *anteiso*-C_15:0_ (12), *iso*-C_15:0_ (9)
Physiological		
Growth at/with:		
15 °C	+	w
37 °C	+++	+
pH 5	w	+
pH 10	+	+++
pH 11	w	+++
7%–15% NaCl	–	++ or +
Gelatin hydrolysis	+	–
Acid production from:		
d-Adonitol	+	–
Amygdalin	w	–
Arbutin	w	–
Esculin ferric citrate	w	–
Gentiobiose	+	–
Gluconate	–	+
d-Maltose	–	+
Methyl-α-d-mannopyranoside	+	–
Salicin	w	–
d-Turanose	–	+
Xylitol	w	+
d-Xylose	–	w
Biochemical		
*N*-Acetyl-β-glucosaminidase	w	+
Chymotrypsin	–	w
β-Galactosidase	+	w
Pyrrolidonyl arylamidase	w	+

* On Trypticase Soy agar. Differences on the other agar media are shown in Table 1; +, positive; –, negative; w, weak.

**Table 3:** Polyketide synthase (PKS) and nonribosomal peptide synthetase (NRPS) in the gene clusters of *Streptomyces* sp. TP-A0598.

Gene Cluster	Predicted Product	Locus Tag (TPA0598_)	Size (aa)	Domain Organization
***t1pks-1***	Unknown	10_00280	2436	KS/AT/KR
		10_00270	1690	KS/AT/DH/ACP
***t1pks-2***	Unknown	04_06320	444	KS
		04_06310	2113	KS/AT/DH/ER/KR/ACP
***t2pks-1***	Spore pigment	03_01500	422	KSα
		03_01510	426	KSβ (CLF)
		03_01520	89	ACP
*t2pks-2* (*oxy*)	Oxytetracycline	07_00590	425	KSα
		07_00600	426	KSβ (CLF)
		07_00610	95	ACP
***t3pks-1*** (***rpp***)	THN	03_03810	353	KS
***nrps-1***	x-Leu-Val-y	07_04820	1066	C/A/T
		07_04810	641	A_(leu)_/T
		07_04800	637	A_(val)_/T
		07_04790	950	C/T
*nrps-2*	x-Asp-Asn-Leu-Phe-Thr-y-Leu	02_01330	863	C/A/T
		02_01450	2734	A_(asp)_/T-C/A_(asn)_/T-C/A_(leu)_/T
		02_01460	2627	C/A_(phe)_/T-C/A_(thr)_/T/E
		02_01470	1883	C/T-C/A_(leu)_/T-TE
*pks/nrps-1*	lydicamycin,TPU-0037-A to -D	03_00740	3598	KS/AT/DH/KR/ACP -KS/AT/DH/KR/ACP
		03_00750	7054	KS/AT/DH/KR/ACP -KS/AT/DH/KR/ACP -KS/AT/DH/KR/ACP -KS/AT/DH/KR/ACP
		03_00760	3548	KS/AT/DH/KR/ACP -KS/AT/DH/KR/ACP
		03_00770	1846	KS/AT/DH/KR/ACP
		03_00780	5648	KS/AT/DH/ER/KR/ACP -KS/AT/DH/KR/ACP -KS/AT/DH/KR/ACP
		03_00790	3662	KS/AT/KR/ACP -KS/AT/DH/ER/KR/ACP
		03_00800	3265	KS/AT/DH/KR/ACP -KS/AT/KR/ACP
		03_00820	1031	C/A/T
		03_00840	1923	ACP-KS/AT/DH/KR/ACP
*pks/nrps-2*	Ser-y-Val-pk	08_01960	556	C/T
		08_01950	1139	T-C/A_(val)_/T
		08_01940	1207	KS/AT/ACP-TE
		08_01890	783	A_(ser)_/T

Gene clusters highlighted in boldface are also present in the genome of *S. angustmyceticus* NBRC 3934^T^. Abbreviations: A, adenylation; ACP, acyl carrier protein; AT, acyltransferase; C, condensation; DH, dehydratase; DHB, dihydroxybenzoate; E, epimerisation; ER, enoylreductase; KR, ketoreductase; KS, ketosynthase; MT, methyltransferase; pk, moiety derived from PKS pathway; T, thiolation; TE, thioesterase; THN, tetrahydroxynaphthalene; x, unidentified amino acid; y, unknown building block because A domain is not present in the module. Predicted substrates of A domains are shown in subscript brackets. The closest homolog of each protein is shown in Table S1.

**Table 4:** PKS and NRPS in the gene clusters of *S. angustmyceticus* NBRC 3934^T^.

Gene Cluster	Predicted Product	Locus Tag (San01_)	Size (aa)	Domain Organization
*****t1pks-1*****	Unknown	16600	2469	KS/AT/KR
		16610	1601	KS/AT/ACP
***t1pks-2***	Unknown	20810	428	KS
		20820	2117	KS/AT/DH/ER/KR/ACP
*t1pks-3* (*tsn*)	Trichostatin A	s29-1 ^t,^*	>375	ACP/KS
		RS35710 ^t^	>471	DH/ACP
		RS35715 ^t^	>897	KS/AT
		RS31690 ^t^	>1500	KS/AT/DH/KR/ACP
		64470	2005	KS/AT/DH/KR/ACP-TE
*t1pks-4*	Caniferolides	s04-1 ^t,^*	>3397	KS/AT/ACP -KS/AT/KR/ACP -KS/AT
		RS35695 ^t^	>990	AT/KR/ACP
		s40-2 ^t,^*	>571	KS
		RS35360 ^t^	>4323	AT/KR/ACP -KS/AT/KR/ACP -KS/AT/DH/KR/ACP
		71810	3953	KS/AT/DH/KR/ACP -KS/AT/DH/ER/KR/ACP
		RS35370 ^t^	>2135	KS/AT/KR/ACP -KS
		RS35705 ^t^	>1815	AT/DH/ER/DH/KR/ACP -KS
		RS35690 ^t^	>1167	AT/KR/ACP
		s39-1 ^t,^*	>577	KS
		RS35595 ^t^	>6115	AT/KR/ACP -KS/AT/KR/ACP -KS/AT/DH/ACP -KS/AT/KR/ACP -KS
		RS31970^t^	>1754	AT/DH/ER/KR/ACP
		65020	5281	KS/AT/KR/ACP -KS/AT/DH/ER/KR/ACP -KS/AT/KR/ACP
		65010	3643	KS/AT/KR/ACP -KS/AT/DH/KR/ACP -TE
		64920	2404	CoL/T-KS/AT/DH/KR/ACP
***t2pks-1***	Spore pigment	26680	422	KSα
		26670	416	KSβ (CLF)
		26660	96	ACP
*t2pks-3*	Trioxacarcin	00550	421	KSα
		00560	417	KSβ (CLF)
		00570	89	ACP
		00580	660	AT
***t3pks-1*** (***rpp***)	THN	24600	354	KS
***nrps-1***	x-Leu-Val-y	06160	1066	C/A/T
		06150	641	A_(leu)_/T
		06140	637	A_(val)_/T
		06130	950	C/T
*nrps-3*	Leu-Ala-x-Ala-Leu-x-Thr -Orn-x-Leu	24170	6209	A_(leu)_/T-C/A_(ala)_/T/E -C/A/T/E-C/A_(ala)_/T/E-C/A_(leu)_/T
		24160	6677	C/A/T/E-C/A_(thr)_/T-C/A_(orn)_/T/E -C/A/T-C/A_(leu)_/T/E
*nrps-4*	mx-x-Gly-	36160	3675	A/MT/T-C/A/T-C/A_(gly)_/T
		s07-1 ^t,^*	>398	C
*nrps-5*	Thr-Thr-x, Arg-Pro	27520 ^t^	>3401	A_(thr)_/T-C/A_(thr)_/T-C/A/T-TE
		27580	591	A_(arg)_/T
		27590	1,446	C/A_(pro)_/T-TD
*pks/nrps-3*	Guadinomine, partial	61570	252	KR
		61560	303	AT
		61550	302	AT
		61530	2393	A_(pip)_/T-KS/AT/DH/KR/ACP
		61520	270	TE
		61190	1178	A/T-TD
*pks/nrps-4*	Ala-Thr-pk	12760	93	ACP
		12770	674	KS/DH
		12780	526	A_(ala)_
		12800	1050	A/T_(thr)_-C
		12810	274	TE

* *Italic*, locus tag is not provided. The open reading frame (ORF) regions in nucleotides are as follows: *s29-1^t^*, BLAG01000032 1-1,125 (+); *s04-1^t^,* BLAG01000007 3-10,193 (-); *s40-2^t^*, BLAG01000043 3,024-4,736 (+); *s39-1^t^*, BLAG01000042 1-1,734 (-); *s07-1^t^*, BLAG01000010 2-1,195 (-). Gene clusters highlighted in boldface are also present in the genome of *Streptomyces* sp. TP-A0598. ^t^ Not completely sequenced because the ORF is present at the terminal of the scaffold sequence. Undetermined domains are shown as ‘…’. Abbreviations are the same as those of Table 3. mx, methyl-amino acid; TD, termination domain. The closest homolog of each protein is shown in Table S2.

## Supplementary Materials

The following are available online at https://www.mdpi.com/2076-2607/8/3/370/s1, Table S1: Closest homolog of PKSs and NRPSs in the gene clusters of *Streptomyces* sp. TP-A0598, Table S2: Closest homolog of PKSs and NRPSs in the gene clusters of *Streptomyces angustmyceticus* NBRC 3934^T^.
