# Supplementary material for: Streptomyces lydicamycinicus sp. nov. and Its Secondary Metabolite Biosynthetic Gene Clusters for Polyketide and Nonribosomal Peptide Compounds
Source: Microorganisms. 2020 Mar 6;8(3):370. doi: 10.3390/microorganisms8030370 (PMC7143880; doi:10.3390/microorganisms8030370)
Supplement: Supplementary file 1 [file microorganisms-08-00370-s001.pdf]

microorganisms

Supplemental Data

Streptomyces lydicamycinicus sp. nov. and its secondary metabolite biosynthetic gene clusters for polyketide and nonribosomal peptide compounds

Hisayuki Komaki [1,*], Akira Hosoyama [1], Yasuhiro Igarashi [2] and Tomohiko Tamura [1]

[1] Biological Resource Center, National Institute of Technology and Evaluation (NBRC); komaki-hisayuki@nite.go.jp, hosoyama-akira@nite.go.jp, tamura-tomohiko@nite.go.jp

[2] Biotechnology Research Center and Department of Biotechnology, Toyama Prefectural University; yas@pu-toyama.ac.jp

* Correspondence: komaki-hisayuki@nite.go.jp; Tel.: +81-438-20-5764

Table S1. Closest homolog of PKSs and NRPSs in the gene clusters of *Streptomyces* sp. TP-A0598 · page 2

Table S2. Closest homolog of PKSs and NRPSs in the gene clusters of *Streptomyces angustmyceticus* NBRC 3934ᵀ · · · · · · · · · · · · · · · · · · page 3

Table S1. Closest homolog of PKSs and NRPSs in the gene clusters of *Streptomyces* sp. TP-A0598.

Gene cluster	Locus tag (TPA0598)	I/S* (%)	Description and accession number of the closest homolog (homolog whose metabolite is experimentally identified)
t1pks-1	_10_00280	91/92	Phenolphthiocerol synthesis polyketide synthase type I Pks15/1, OSY45424
	_10_00270	86/87	Phthioceranic/hydroxyphthioceranic acid synthase, OSY45423
t1pks-2	_04_06320	99/99	3-oxoacyl-[acyl-carrier-protein] synthase 2, OSY38813
	_04_06310	98/98	Phthioceranic/hydroxyphthioceranic acid synthase, OSY38814
t2pks-1	_03_01500	99/100	β-ketoacyl-[acyl-carrier-protein] synthase family protein, QEV50971
	_03_01510	99/99	ketosynthase chain-length factor, QEV50970
	_03_01520	100/100	acyl carrier protein, QEV50969
t2pks-2 (*oxy*)	_07_00590	93/95 (88/93)	Actinorhodin polyketide putative β-ketoacyl synthase 1, OSY48324 (OxyA, AAZ78325)
	_07_00600	86/90 (77/84)	Actinorhodin polyketide putative β-ketoacyl synthase 2, OSY48325 (OxyB, AAZ78326)
	_07_00610	83/90 (70/80)	Oxytetracycline polyketide synthase acyl carrier protein, OSY48326 (OxyC, AAZ78327)
t3pks-1 (*rpp*)	_03_03810	98/98	1,3,6,8-tetrahydroxynaphthalene synthase, GFE29002
nrps-1	_07_04820	97/98	non-ribosomal peptide synthetase, WP_085926581
	_07_04810	94/95	non-ribosomal peptide synthetase, WP_085926580
	_07_04800	96/96	non-ribosomal peptide synthetase, WP_085926605
	_07_04790	95/96	NAD-dependent epimerase/dehydratase family protein, WP_085926579
nrps-2	_02_01330	95/97	Tyrocidine synthase 3, OSY47214
	_02_01450	97/98	hypothetical protein Srufu_42150, GFE31835
	_02_01460	95/97	hypothetical protein Sgleb_38680, GFE15821
	_02_01470	96/97	non-ribosomal peptide synthetase, WP_085923271
pks/nrps-1	_03_00740	95/96	polyketide synthase, GFE28620
	_03_00750	93/95	hypothetical protein Srufu_10010, GFE28621
	_03_00760	94/96	type I polyketide synthase, WP_085923489
	_03_00770	92/95	polyketide synthase, AZS70416
	_03_00780	95/96	type I polyketide synthase, WP_085923487
	_03_00790	96/97	type I polyketide synthase, WP_085923486
	_03_00800	97/97	hypothetical protein Srufu_10060, GFE28626
	_03_00820	97/97	hypothetical protein Srufu_10080, GFE28628
	_03_00840	95/96	hypothetical protein Srufu_10100, GFE28630
pks/nrps-2	_08_01960	82/88	condensation protein, WP_107065154
	_08_01950	80/87	non-ribosomal peptide synthetase, WP_031138763
	_08_01940	80/85	type I polyketide synthase, WP_078904507
	_08_01890	76/82	amino acid adenylation domain-containing protein, WP_107066826

*I/S, identity/similarity in amino acid sequence.

Table S2. Closest homolog of PKSs and NRPSs in the gene clusters of *Streptomyces angustmyceticus* NBRC 3934^{T}

Gene cluster	Locus tag (San01_)	I/S* (%)	Description and accession number of the closest homolog (homolog whose metabolite is experimentally identified)
*t1pks-1**	16600	83/85	type I polyketide synthase, WP_093491222
	16610	85/86	hypothetical protein Scani_28540, GFE06586
*t1pks-2**	20810	95/97	ketoacyl synthase, WP_088797712
	20820	92/95	type I polyketide synthase, WP_093492590
t1pks-3 (*tsn*)	*s29-1*[t]	97/98	type I polyketide synthetase (TsnB1), BAX57200
	RS35710[t]	94/96	type I polyketide synthetase (TsnB1), BAX57200
	RS35715[t]	95/97	type I polyketide synthetase (TsnB2), BAX57201
	RS31690[t]	95/96	type I polyketide synthetase (TsnB2), BAX57201
	64470	95/97	type I polyketide synthetase (TsnB3), BAX57202
t1pks-4	*s04-1*[t]	87/90	type I polyketide synthase (ScaP1), QBF51754
	RS35695[t]	87/92	SDR family NAD(P)-dependent oxidoreductase, TXD00034
		(86/90)	(type I polyketide synthase (ScaP1), QBF51754)
	s40-2[t]	94/96	Acyl transferase domain-containing protein, SOE09197
		(94/97)	(type I polyketide synthase (ScaP2), QBF51755)
	RS35360[t]	89/92	SDR family NAD(P)-dependent oxidoreductase, TXD00033
		(89/92)	(type I polyketide synthase (ScaP2), QBF51755))
	71810	88/91	Type I polyketide synthase, CUW01173
		(87/90)	(type I polyketide synthase (ScaP3), QBF51756)
	RS35370[t]	91/93	type I polyketide synthase, WP_093488014
		(89/92)	(type I polyketide synthase (ScaP4), QBF51757)
	RS35705[t]	89/92	SDR family NAD(P)-dependent oxidoreductase, TXD00026
		(85/90)	(type I polyketide synthase (ScaP4), QBF51757)
	RS35690[t]	86/89	type I polyketide synthase (ScaP4), QBF51757
	s39-1[t]	94/97	type I polyketide synthase (ScaP5), QBF51758
	RS35595[t]	89/92	type I polyketide synthase (ScaP5), QBF51758
	RS31970[t]	90/93	hypothetical protein Stube_65570, GFE41884
		(90/93)	(type I polyketide synthase (ScaP5), QBF51758)
	65020	99/100	SDR family NAD(P)-dependent oxidoreductase, TXD00025
		(89/92)	(type I polyketide synthase (ScaP6), QBF51759)
	65010	92/94	hypothetical protein Stube_65590, GFE41886
		(90/92)	(type I polyketide synthase (ScaP7), QBF51760)
	64920	88/92	AMP-binding protein, TXD00261
		(87/91)	(type I polyketide synthase (ScaP8), QBF51769)
*t2pks-1**	26680	97/99	β-ketoacyl-[acyl-carrier-protein] synthase family protein, WP_030983599
	26670	99/99	ketosynthase chain-length factor, WP_088796350
	26660	96/97	putative acyl carrier protein, GFE08130
t2pks-3	00550	99/99	actinorhodin polyketide putative β-ketoacyl synthase 1, GFE06353
		(86/92)	(TxnA1, AKT74262)
	00560	96/97	actinorhodin polyketide putative β-ketoacyl synthase 1, GFE06352
		(78/87)	(TxnA2, AKT74261)
	00570	98/98	actinorhodin polyketide synthase acyl carrier protein, GFE06351
		(73/85)	(TxnA3, AKT74260)
	00580	87/88	hypothetical protein Scani_26180, GFE06350
		(63/70)	(TxnA4, AKT74259)
t3pks-1 (*rpp*)*	24600	97/98	1,3,6,8-tetrahydroxynaphthalene synthase, GFE07918
*nrps-1**	06160	92/95	non-ribosomal peptide synthetase, WP_088801550
	06150	87/90	non-ribosomal peptide synthetase, WP_129297982
	06140	90/92	amino acid adenylation domain-containing protein, SED80324
	06130	88/91	non-ribosomal peptide synthetase/polyketide synthase, KOG49533
nrps-3	24170	91/93	amino acid adenylation domain-containing protein, WP_129292551
	24160	91/93	non-ribosomal peptide synthetase, WP_093640679
nrps-4	36160	87/91	amino acid adenylation domain-containing protein, TXS70023

	s07-1[t]	93/96	non-ribosomal peptide synthetase, partial, TXS70022
nrps-5	27520[t]	90/93	amino acid adenylation domain-containing protein, TXS81310
	27580	85/88	amino acid adenylation domain-containing protein, TXS81304
	27590	89/92	amino acid adenylation domain-containing protein, TXS81303
pks/nrps-3	61570	82/89	SDR family NAD(P)-dependent oxidoreductase, WP_138902144
	61560	87/94	ACP S-malonyltransferase, MQS98670
	61550	81/88	hypothetical protein, MQS98671
	61530	82/88	amino acid adenylation domain-containing protein, WP_138902148
		(54/64)	(mixed NRPS PKS (GdnE), AFU82614)
	61520	74/82	thioesterase, WP_138902149
	61190	89/91	AMP-binding protein, WP_100605862
pks/nrps-4	12760	74/76	acyl carrier protein, WP_058042275
	12770	84/88	polyketide synthase, WP_058042274
	12780	88/93	amino acid adenylation domain-containing protein, WP_058042273
	12800	83/87	non-ribosomal peptide synthetase, WP_058042271
	12810	77/83	thioesterase, WP_058042270

*I/S, identity/similarity in amino acid sequence.

© 2020 by the authors. Submitted for possible open access publication under the terms and conditions of the Creative Commons Attribution (CC BY) license (http://creativecommons.org/licenses/by/4.0/).
